# Warm-water decapods and the trophic amplification of climate in the North Sea

**DOI:** 10.1098/rsbl.2010.0394

**Published:** 2010-06-16

**Authors:** J. A. Lindley, G. Beaugrand, C. Luczak, J.-M. Dewarumez, R. R. Kirby

**Affiliations:** 1 Sir Alister Hardy Foundation for Ocean Science, Plymouth, UK; 2 Centre National de la Recherche Scientifique, LOG UMR 8187, France; 3 School of Marine Science and Engineering, University of Plymouth, Drake Circus, Plymouth PL4 8AAUK

**Keywords:** benthos, bivalves, flatfish, *Polybius henslowii*, range, temperature

## Abstract

A long-term time series of plankton and benthic records in the North Sea indicates an increase in decapods and a decline in their prey species that include bivalves and flatfish recruits. Here, we show that in the southern North Sea the proportion of decapods to bivalves doubled following a temperature-driven, abrupt ecosystem shift during the 1980s. Analysis of decapod larvae in the plankton reveals a greater presence and spatial extent of warm-water species where the increase in decapods is greatest. These changes paralleled the arrival of new species such as the warm-water swimming crab *Polybius henslowii* now found in the southern North Sea. We suggest that climate-induced changes among North Sea decapods have played an important role in the trophic amplification of a climate signal and in the development of the new North Sea dynamic regime.

## 1. Introduction

Several studies have described pronounced and sustained responses of marine ecosystems to climate warming (Intergovernmental Panel on Climate Change WGII 2007). We have recently proposed a new mechanism of ecosystem regime change called trophic amplification, where linkages in the food web amplify a climate signal leading rapidly to a new dynamic regime (Kirby & Beaugrand 2009). Our mechanism is based upon an examination of change in the North Sea plankton, which during the 1980s experienced a change in hydroclimatic forcing that caused a rapid temperature-driven ecosystem shift (Beaugrand *et al*. 2008). Changes in the North Sea after the ecosystem shift include an increase in phytoplankton, a change in the composition and abundance of the holozooplankton (animals that spend their whole life in the plankton), increases in the frequency of jellyfishes and in the abundance of decapod and echinoderm larvae, and a decrease in bivalve larvae and flatfish recruits (Kirby & Beaugrand 2009).

We have suggested that decapods in particular are an important trophic group in propagating the hydroclimatic signal through the North Sea food web (Kirby & Beaugrand 2009); by top-down effects, decapods can influence the holozooplankton (Frank *et al*. 2005; Signa *et al*. 2008) and the recruitment of both bivalves (Barkai & McQuaid 1988; Beukema & Dekker 2005), and flatfishes (van der Veer & Bergman 1987). Consequently, we argued that climate-induced changes in predator–prey interactions could explain why bivalve larval abundance has declined steadily since the end of the 1990s, as decapods have increased in number (Kirby & Beaugrand 2009); this despite the fact the abundance of both bivalve and decapod larvae correlates positively with annual changes in the surface temperature of North Sea, which has warmed by 1°C since the mid-1980s.

The changes in the North Sea plankton have been seen in continuous plankton recorder (CPR) samples. Unfortunately, decapods (with the exception of sergestids) are recorded only in occasional finer taxonomic resolution studies (Rees 1952, 1955; Lindley 1987; Lindley *et al*. 1993), and the most abundant decapod larvae in North Sea CPR samples, most Polybiinae (swimming crabs), cannot be identified specifically by morphology. The detailed nature of the changes in the Decapoda, which may be due either to an increase in abundance of existing taxa and/or the colonization of new species, are therefore unclear.

Here, we re-examined North Sea CPR samples collected in 2008 (the most recent year for which samples were available) to identify the dominant Decapoda to species or generic level. We then compared changes in decapod and bivalve larval abundance over the period 1958–2007 with changes in their benthic abundance, determined from macrobenthic samples collected at Gravelines in the southern North Sea between 1978 and 2007.

## 2. Material and methods

### (a) Decapod larvae

Decapod larvae were collected during 2008 by the CPR survey, which has operated on a monthly basis in the North Sea since 1946 (Batten *et al*. 2003). Decapod larvae, with the exception of most Polybiinae that cannot be identified visually, were identified by morphology to the finest taxonomic resolution possible (Lindley 1987). To identify larval Polybiinae, we amplified and sequenced partial mtDNA 16S rDNA gene sequences from individual larvae following standard protocols (Kirby *et al*. 2006). The identity of the amplification products was then determined by comparison to a mtDNA 16S rDNA sequence database compiled from the following adult species: *Liocarcinus arcuatus*, *Liocarcinus corrugatus*, *Liocarcinus depurator*, *Liocarcinus holsatus*, *Liocarcinus marmoreus*, *Liocarcinus pusillus*, *Macropipus tuberculatus*, *Necora puber* and *Polybius henslowii* (GenBank accession numbers, GQ268539–GQ268547).

### (b) Benthic data

A total of 10 benthic samples were collected at a 10 m depth on four to six occasions a year between 1978 and 2007 at Gravelines in the southern North Sea (51°01′ 40 N, 2°04′ 35 E). Samples were preserved in 4–5% neutralized formaldehyde prior to the identification of decapods and bivalves to species level and their numeration (Ghertsos *et al*. 2000).

### (c) Statistical methods

The mean monthly number of each decapod taxon per CPR sample was determined for a 1° latitude by 2° longitude grid and used to calculate abundance in six North Sea standard areas (electronic supplementary material, figure S1). The mean abundance (mean of monthly values) (Colebrook & Robinson 1965) was then calculated for area B2, which is influenced strongly by Atlantic inflow around the North of Scotland, and for the average of the five remaining areas (Lindley *et al*. 1993). We then compared our results with our previous studies in 1981–1983 and 1989 (Lindley 1987; Lindley *et al*. 1993), and those of Rees (1955).

To compare changes in decapod and bivalve larvae in the plankton, and the plankton with the benthos, we interpolated the data from 54 507 CPR samples, after applying the transformation log_10_(*x* + 1), on a 1° latitude by 1° longitude grid over the spatial domain 3.5° W–9.5° E and 50.5° N–60.5° N using an inverse-squared distance method with a 250 km search radius (Beaugrand *et al*. 2000). Then, to determine the extent of the change between decapod and bivalve larvae we compared the periods 1958–1979 and 1990–2007; periods before and after the regime shift (Beaugrand *et al*. 2008). Maps of each year were averaged for each period and the magnitude of change between the two periods was calculated by subtracting the first map from the second. A Kruskall–Wallis test, applied to each geographical cell between years of the two periods, assessed the probability of change.

To compare the plankton data with the benthic data, we performed a spatialized standardized principal component analysis on the table, year (1958–2007) × geographical cells of the abundance of both decapods and bivalves. Correlation analysis between the first principal component and the benthic data was then performed with the probability of each correlation coefficient corrected to account for temporal autocorrelation (Pyper & Peterman 1998).

## 3. Results

**Figure 1.**
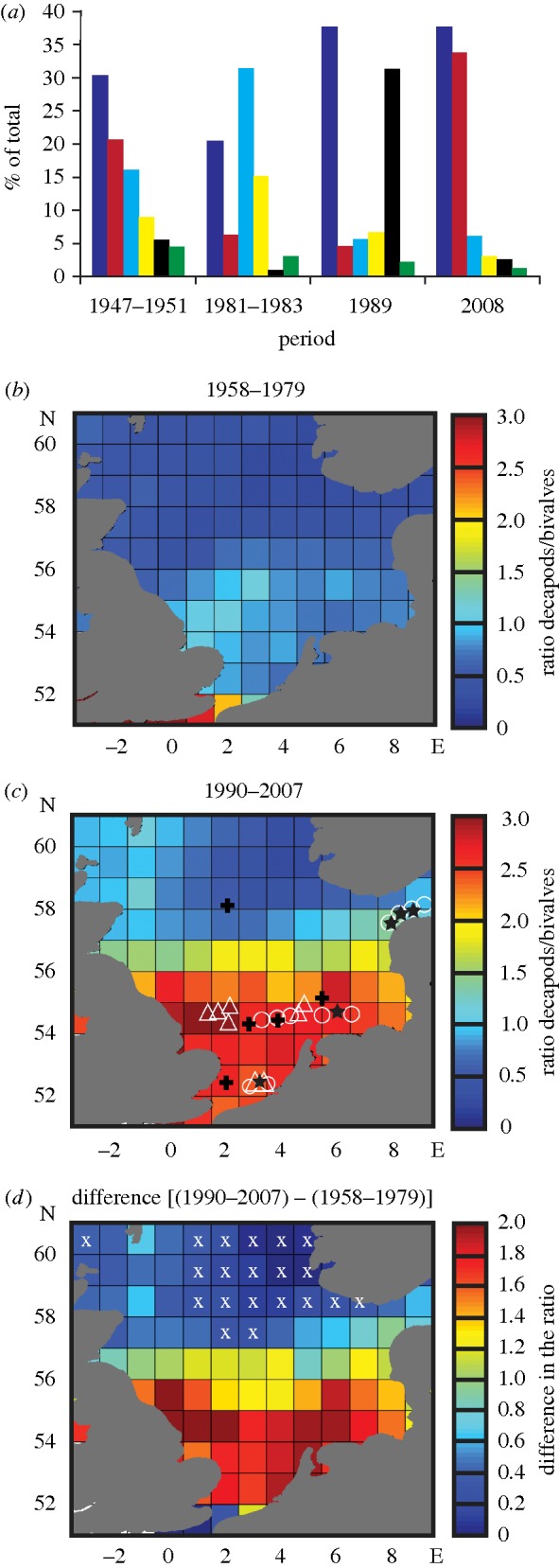
Long-term changes in decapod and bivalve larvae. (*a*) The most abundant taxa identified in CPR samples during 2008 compared with our data from 1981–1983, 1989 and that of Rees (1955). (*b,c*) Spatial changes in the ratio decapods/bivalves for the periods 1958–1979 and 1990–2007. (*d*) Difference in the ratio between the two periods; crosses indicate where the difference is not significant. (*a*) Dark blue bars, *Polybiinae* spp.; yellow bars, *Cancer pagurus*; red bars, *Upogebia deltaura*; black bars, *Pisidia longicornis*; blue bars, *Callianassa subterranea*; green bars, *Pagurus berhardus*. (*c*) Triangles, *Polybius henslowii*; circles, *Thia scutellata*; crosses, *Goneplax rhomboides*; stars, *Pestarella tyrrhena*.

The most abundant decapod taxa in CPR samples during 2008 were the larvae of the Polybiinae and the mud shrimps *Upogebia deltaura* and *Callianassa subterranea*; these were also the most abundant species between 1947 and 1951 (Rees 1955) (electronic supplementary material, table S1). Figure 1*a* compares our data from 1981–1983, 1989 and 2008 with that of Rees (1955). We observed the greatest change in the ratio decapod/bivalve larvae occurred in the southern North Sea where the ratio doubled between the two periods (figure 1*b–d*). The change in the ratio was significant throughout the North Sea except for the area close to the Norwegian Trench (figure 1*d*).

The most notable occurrence among the decapod larvae was the warm-water swimming crab *P. henslowii* (figure 1*c* and electronic supplementary material, table S2), which had not been recorded previously in the North Sea except in Shetland waters (Ingle 1980), and subsequently only exceptionally east of Scotland and off southern Norway (Udekem d'Acoz 1999). The presence of three further taxa in CPR samples in 2008 was also noteworthy (figure 1*c*). The crab *Goneplax rhomboides* was unrecorded in the North Sea (Ingle 1980; apart from a single record off Northumberland in 1963 noted by Moore 1986), although it was found in Swedish waters recently (Berggren 2008). The two warm-water species, *Thia scutellata* and the commercially important mud shrimp *Pesterella tyrrhena*, were also more widespread than before (Lindley 1987). Finally, while Crangonidae were less abundant in CPR samples, we found changes in the abundance and proportions of these commercially important species (electronic supplementary material, table S3).

**Figure 2.**
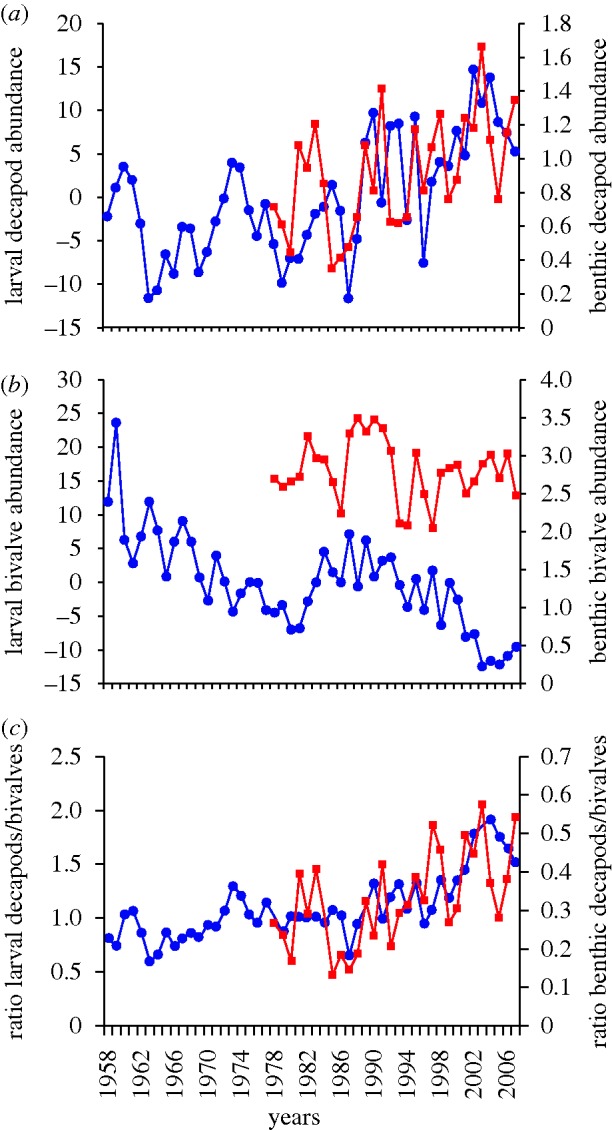
Long-term changes in the North Sea plankton and the benthos at Gravelines. (*a*) Decapod abundance. (*b*) Bivalve abundance. (*c*) Long-term change in the ratio decapod/bivalve abundance. (*a*,*b*) Blue circles, planktonic larvae; red squares, benthic adults (*c*) Blue circles, planktonic decapods/bivalves; red squares, benthic decapods/bivalves.

Long-term changes in the North Sea plankton and the benthos at Gravelines were positively correlated for decapods (*r* = 0.446, *p* = 0.048; figure 2*a*). No similar correlation was detected for bivalves (*r* = 0.266, *p* = 0.26; figure 2*b*). The correlation between the ratios calculated between the plankton and the benthic data was positive (*r* = 0.525 *p* = 0.065; figure 2*c*). However, the correction for temporal autocorrelation affected strongly the probability (*p* = 0.0029), indicating a strong autocorrelation in the time series.

## 4. Discussion

The most abundant decapod larvae in CPR samples are the Polybiinae and in 2008, *P. henslowii*, and *L. depurator* were the dominant species. The swimming crab *P. henslowii*, which as an adult has only been recorded in the North Sea rarely, along with larvae of the angular crab *G. rhomboides*, which was not reported in earlier studies of CPR samples, are both warm-water species. Together with a range expansion of the warm-water *P. tyrrhena* and *T. scutellata*, the distributions of these four taxa in 2008 (figure 1*c*) indicate an increase in abundance and range of warm-water decapods in the North Sea.

The spatial change in the ratio decapods/bivalves in the plankton was greatest in the shallower southern North Sea and German Bight, the coldest areas of the North Sea in winter (Lee & Ramster 1981), and to the North of Denmark (figure 1*d*). All these regions are now warmer in winter than previously (Kirby *et al*. 2007). With one exception (a single *G. rhomboides* larva in the northern North Sea) the appearance of new warm-water decapod taxa coincided with those areas where the change in the ratio is greatest (figure 1*c,d*). The boundary of the region of greatest change in the ratio decapod/bivalve larvae follows approximately the mixed stratified environment of the North Sea (Pingree & Griffiths 1978).

The strong decrease in the corrected probability between the ratio decapods/bivalves in the North Sea plankton and decapods/bivalves in the benthos at Gravelines (figure 2*c*) observed from the non-corrected probability, revealed that the correlation is mainly related to low-frequency variability (i.e. long-term change) and pseudocyclic variation. Long-term change in the ratio (figure 2*c*) reflects an increase in the proportion of decapods to bivalves in both the North Sea plankton and the benthos at Gravelines. This change, which started during the mid 1980s, was correlated highly with the increase in sea surface temperature and could reflect the nonlinear effect of increased decapod predation upon bivalves (Kirby & Beaugrand 2009). In this regard, the increased abundance and proportion of Crangonidae in the plankton since the 1980s is noteworthy (electronic supplementary material, table S3); these commercially important brown shrimps are important bivalve predators (Beukema & Dekker 2005).

Our study suggests that a component of the increase in decapod larvae in the North Sea is a greater abundance and range of warm-water taxa. In this regard, the occurrence of the opportunistic carnivore, *P. henslowii* is especially notable. Although it is a benthic species, *P. henslowii* has periodic pelagic phases, which is unusual for crabs, and it can swarm in large numbers when conditions are favourable exhibiting range shifts in response to hydroclimatic change (Signa *et al*. 2008).

In addition to being important predators of bivalves, predation by decapods on the seabed may have contributed to the decline of flatfish recruits and predation by decapod larvae and adult swimming crabs during their pelagic phase may influence the zooplankton. In this way, the increase in warm-water decapods may be a key component of the trophic amplification of hydroclimatic change and the development of the new North Sea ecosystem dynamic regime.
